# CCL14, identified by multi-omics approach, serves as a novel indicator of disease severity and progression in lymphangioleiomyomatosis

**DOI:** 10.1186/s13023-025-04193-2

**Published:** 2026-01-20

**Authors:** Wenxue Bai, Lijuan Hua, Xuezhao Wang, Mengyao Guo, Lirong Chen, Bingyi Liu, Yi Wang, Ying Zhou, Qi Wang, Ni Zhang, Min Xie

**Affiliations:** 1 Department of Respiratory and Critical Care Medicine, Tongji Hospital, Tongji Medical College, Huazhong University of Science and Technology, Wuhan, 430030 China; 2 Department of Thoracic Surgery, Tongji Hospital, Tongji Medical College, Huazhong University of Science and Technology, Wuhan, 430030 China; 3 Department of Emergency and Critical Care Medicine, Tongji Hospital, Tongji Medical College and State Key Laboratory for Diagnosis and Treatment of Severe Zoonotic Infectious Disease, Huazhong University of Science and Technology, Wuhan, 430030 China

## Abstract

**Background:**

Lymphangioleiomyomatosis (LAM) is a rare, multisystemic metastatic disease. Chemokines are implicated in promoting LAM cell migration and tumor progression. Our prior plasma proteomics identified elevated C-C motif chemokine ligand 14 (CCL14) in LAM, yet its role remains unexplored.

**Methods:**

Proteomic analysis identified CCL14 as differentially expressed in LAM patients versus healthy controls. Single-cell RNA sequencing (scRNA-seq) of lung tissues (six LAM patients, five healthy donors) identified the cellular source of CCL14 and explored its functional pathways bioinformatically. ELISA-quantified plasma CCL14 levels were analyzed for correlations with clinical phenotypes and longitudinal disease progression in 53 LAM patients and 25 controls.

**Results:**

Proteomics and scRNA-seq revealed upregulation of CCL14 in LAM patients, primarily localized to lymphatic and vascular endothelial cells. Functional enrichment linked CCL14 to proliferative (mTORC1, MYC), inflammatory (TNF-α/NF-κB), and chemotactic pathways. CellPhoneDB indicated CCL14 mediates interactions between endothelial cells and innate immune/alveolar epithelial cells, and between endothelial cells themselves, via ACKR2, CCR3 and CCR1. Clinically, plasma CCL14 levels were significantly elevated in LAM patients versus controls (*p* = 0.003). Subgroup analyses demonstrated higher CCL14 levels in patients with angiomyolipomas (AMLs) versus those without, and in CT grade III versus grade I/II. Critically, CCL14 predicted disease progression: baseline CCL14 levels were higher in progressive versus stable patients (*p* = 0.0266) and positively correlated with annual increase in the percentage of cystic lung volume (*r* = 0.4051, *p* = 0.0446).

**Conclusions:**

CCL14 is a critical regulatory molecule within the LAM microenvironment and a promising biomarker for disease severity and progression.

**Supplementary information:**

The online version contains supplementary material available at 10.1186/s13023-025-04193-2.

## Background

Lymphangioleiomyomatosis (LAM) is a rare, progressive multisystem disorder predominantly affecting women of childbearing age. It is characterized by the abnormal proliferation of smooth muscle-like LAM cells, which drive the cystic destruction of lung parenchyma, lymphatic complications (such as chylous effusions and lymphangioleiomyomas), and renal angiomyolipomas (AMLs) [1]. Despite the central pathogenic role of *TSC1/TSC2* gene mutations and consequent mTOR pathway hyperactivation is well-established, clinical management remains challenged by heterogeneous progression, variable treatment responses and insufficiency of biomarkers in prognostic prediction [2, 3]. Current biomarkers such as serum vascular endothelial growth factor D (VEGF-D), while valuable, exhibits limitations in outcome prediction, monitoring disease progression, predicting treatment response, and coverage of all LAM subtypes [1, 4–7]. Consequently, complementary biomarkers are essential to optimize clinical management.

The tumor microenvironment (TME) in LAM exhibits complex interactions between LAM cells, fibroblasts, endothelial cells, epithelial cells and immune populations, which collectively promote matrix remodeling and cyst formation [8–12]. Chemokines, as critical mediators of intercellular communication, display context-dependent “double-edged sword” effects within tumors through ligand-receptor signaling networks, which modulate tumor immunity, angiogenesis, lymphangiogenesis, invasion and metastasis [13–15]. Notably, our prior quantitative proteomic analysis identified a significant elevation of C-C motif chemokine ligand 14 (CCL14) in the plasma of LAM patients [16]. Importantly, CCL14 has been reported to exert divergent functions across different cancer types: in hepatocellular carcinoma, it acts as a tumor suppressor by inhibiting Wnt/β-catenin signaling, with high expression linked to better outcomes [17, 18], while in gastric cancer, low CCL14 expression is significantly associated with prolonged overall survival [19]. Nevertheless, in the context of LAM, the cellular origins, functional roles, and clinical relevance of CCL14 remain unexplored.

This study aimed to evaluate the role of CCL14 in LAM. Using single-cell RNA sequencing (scRNA-seq) data from human LAM lung tissues, we first identified the cellular sources of CCL14. Subsequent bioinformatic analyses elucidated its biological functions and constructed CCL14-mediated intercellular crosstalk networks. Finally, plasma CCL14 levels were validated in a LAM cohort and assessed for associations with clinical phenotypes and disease prognosis.

## Methods

### Study population and sample collection

#### Cohort establishment

A cohort of 53 female LAM patients and 25 matched healthy controls was recruited from our institution between June 2018 and June 2025. The diagnosis of LAM was established according to the definitive diagnostic criteria outlined in the American Thoracic Society/Japanese Respiratory Society (ATS/JRS) Clinical Practice Guideline [20]. Baseline assessment included age, BMI, menopausal status, pulmonary function tests, chest computed tomography (CT) images, and systematic evaluation of LAM-associated manifestations: pneumothorax, chylous effusion, renal AMLs, retroperitoneal lymphangioleiomyoma, and pulmonary hypertension (assessed via echocardiography). The severity of cystic changes on chest CT scans was graded on a scale of I to III, quantified by the extent of pulmonary involvement as follows: grade I ( < one-third), grade II (one-third to two-thirds), and grade III ( > two-thirds) [21]. Longitudinal follow-up occurred at 6–12 month intervals with identical parameters recorded. For quantitative analysis of cystic changes in the lungs, the volume proportion of cystic lesions on chest CT scans was calculated using 3D Slicer software (v5.8.1). Disease progression was comprehensively defined as meeting ≥1 of the following criteria: annual FEV_1_ decline > 30 mL, DLCO decline > 0.24 mmol/min/kpa, DLCO/VA decline relative to baseline > 5%, recurrent pneumothorax (≥3 episodes/year) or refractory pneumothorax, refractory or progressive chylous effusion, renal AMLs growth ≥ 2 mm/year, retroperitoneal mass enlargement ≥ 2 mm/year, newly developed pulmonary hypertension. Patients not fulfilling these criteria were classified as stable.

Lung tissue samples were retrospectively obtained from the Tongji Hospital, Tongji Medical College, Huazhong University of Science and Technology between 2019 and 2025. The LAM group consisted of samples from seven patients with a pathological diagnosis of LAM, who underwent diagnostic open lung biopsy, surgical intervention for pneumothorax, or lung transplantation. The control group included six samples from the adjacent normal parenchyma (at least 3 cm distant from the tumor margin) of lung cancer patients.

This study was approved by the Ethical Research Committee of the Tongji Hospital, Tongji Medical College, Huazhong University of Science and Technology (IRB number: 20211265). Written informed consent was acquired from every participant enrolled in the study.

#### Biological sample collection

Peripheral venous blood samples were collected from all participants using vacuum blood collection tubes. Samples were centrifuged at 1500 × g for 15 min at 4 °C. The supernatant plasma and serum fractions were stored at −80 °C until subsequent analysis. Fresh lung tissues were obtained from two female LAM patients undergoing bullectomy for recurrent pneumothorax. Both patients were mammalian target of rapamycin (mTOR) inhibitor-naïve at surgery. Immediately following resection, tissues were immersed in ice-cold phosphate-buffered saline (PBS) and transferred to the research laboratory for subsequent single-cell isolation procedures.

#### Proteomic analysis

A subset of 10 LAM patients and 6 healthy controls underwent plasma proteomic profiling. Details of this proteomics cohort have been previously published [16].

### Single-cell RNA sequencing (scRNA-seq)

#### Single-cell isolation and sequencing library construction

Fresh tissue samples were enzymatically dissociated into single-cell suspensions using the SeekMate Tissue Dissociation Reagent Kit A Pro (SeekGene, China), performed according to the manufacturer’s standardized protocol. Following dissociation, red blood cells were selectively lysed using an erythrocyte removal solution (Solarbio, China). Cell concentration and viability were then quantitatively assessed employing dual-fluorescence nucleic acid staining with Acridine Orange (AO)/Propidium Iodide (PI) on a Fluorescence Cell Analyzer (Countstar Rigel S2). The resulting viable cell population was washed twice in RPMI-1640 medium (Gibco, USA), then resuspended at a density of 1 × 10^6^ cells/mL in RPMI-1640 supplemented with 2% fetal bovine serum (FBS; Gibco, USA). Single-cell RNA sequencing libraries were constructed using the SeekOne Digital Droplet Single Cell 3’ library preparation kit (SeekGene, China), leveraging its droplet-based partitioning technology for precise single-cell barcoding and cDNA synthesis. Completed libraries underwent high-throughput paired-end sequencing (150 bp read length, PE150) on an Illumina NovaSeq 6000 platform.

#### scRNA-seq data processing

Newly generated raw sequencing data were processed using Fastp (v0.20.1) to trim adapter sequences and remove low-quality bases. Processed reads were aligned to the human reference genome GRCh38 using SeekOne Tools pipeline to generate gene expression matrices. Publicly available datasets for LAM specimens (GSE190260: samples GSM5718714, GSM5718715; GSE135851: samples GSM4035465, GSM4035467) and healthy controls (GSE135851: sample GSM4035472; GSE139819: samples GSM4143262, GSM4143263; GSE122960: samples GSM3489182, GSM3489187) were acquired from GEO database. All datasets were integrated and processed using Seurat (v4.0.0), with cells filtered based on quality thresholds: min.cells ≥ 3, 200 < nFeature_RNA < 8000, nCount_RNA > 300 and percent.mt < 10%. Expression data were log-normalized, followed by identification of 2000 highly variable genes via FindVariableFeatures. Feature expression values underwent z-score standardization (mean-centered with unit variance) prior to dimensionality reduction through principal component analysis (PCA), retaining the first 30 principal components based on elbow plot evaluation. Harmony integration was implemented to address technical batch heterogeneity across datasets. FindClusters was used to cluster cells using the 30 dims at a resolution of 0.5. Finally, transcriptional landscapes were visualized via t-distributed Stochastic Neighbor Embedding (t-SNE).

#### Bioinformatics analysis

Differentially expressed genes (DEGs) were identified using the presto function with selection thresholds of adjusted *p*-value < 0.05 and |log2 fold-change| > 0.25. Subsequent Functional enrichment analysis of DEGs was conducted using Over-Representation Analysis (ORA) in clusterProfiler R package. This examined enrichment in: Gene Ontology biological processes (GO-BP); Hallmark gene sets from MSigDB. Significant terms were defined by a false discovery rate (FDR) < 0.05 with minimum gene set size = 10. CellPhoneDB Python package (v2.1.7) was used to detect ligand-receptor interactions and predict communications among distinct cell populations. Metastasis and inflammation gene sets were sourced from CancerSEA database, while T-cell inhibitory signature was obtained from reference PMID:33711273.

### Immunohistochemical (IHC)

IHC for CCL14 was performed on paraffin-embedded tissue sections. Briefly, after deparaffinization, rehydration, and antigen retrieval, endogenous peroxidase was blocked with 3% H_2_O_2_. For primary antibody staining, sections were incubated overnight at 4 °C with an anti-CCL14 antibody (14216–1-AP, Proteintech). After washing with PBS, a horseradish peroxidase (HRP)-conjugated secondary antibody was incubated with the sections for 1 hour at room temperature. Signal was developed using a diaminobenzidine (DAB) substrate kit (Bosterbio, Wuhan, China), and the reaction was monitored microscopically before being stopped in distilled water. Finally, the sections were counterstained with hematoxylin, dehydrated through graded alcohols, cleared in xylene, and mounted with a synthetic resin.

### Cell culture

Human Umbilical Vein Endothelial Cells (HUVECs) were friendly provided by Department of General Surgery, Tongji Hospital and were grown in Dulbecco’s modified Eagle’s medium (DMEM) containing 10% fetal bovine serum (Wisent, Canada) and 1% penicillin/streptomycin, and incubated at 37 °C in a 5% CO2 humidified incubator.

### Western blot

After treating HUVECs with or without CCL14 (10 or 20 ng/mL) for 24 hours, cells were collected and lysed on ice for 30 minutes using RIPA lysis buffer (Aspenbio, Wuhan, China) containing protease inhibitors. Equal amounts of protein from each sample were separated by 10% SDS-PAGE and transferred to PVDF membranes. The membranes were blocked with 5% non-fat milk and subsequently incubated overnight at 4 °C with specific primary antibodies, followed by a 1-hour incubation with corresponding secondary antibodies at room temperature. The primary antibodies used were as follows: P-S6 (4858T, CST), SERBP2 (28212–1-AP, Proteintech), and β-actin (66009–1-Ig, Proteintech). Protein band intensities were quantified using ImageJ software.

### ELISA analysis of VEGF-D and CCL14

Serum concentrations of VEGF-D in samples from 53 disease subjects and 25 healthy controls were quantified using a human VEGF-D ELISA kit (R&D Systems, USA). Plasma concentrations of CCL14 in the same cohort were measured using a human CCL14 ELISA kit (Boster Biological Technology, China). All ELISA procedures were performed in accordance with the manufacturers’ detailed protocols.

### Statistical analysis

Statistical analyses were performed in GraphPad Prism 9.0 or SPSS 26.0. Intergroup comparisons employed the non-parametric Mann-Whitney U test or Pearson’s chi-square test. Bivariate correlations were assessed using Spearman’s rank correlation coefficient with 95% confidence intervals. The predictive accuracies of CCL14 and VEGF-D for disease progression were evaluated using receiver operating characteristic (ROC) curve analysis. Survival analysis was performed with the Kaplan-Meier method, and differences in disease progression-free survival between groups were compared with the log-rank test. Statistical significance was defined as *p* < 0.05. Data are presented as median with interquartile range (IQR; 25th-75th percentiles) or mean ± standard error of mean (SEM).

## Results

### Proteomic and scRNA-seq analysis reveal elevated CCL14 expression in LAM

**Fig. 1:**
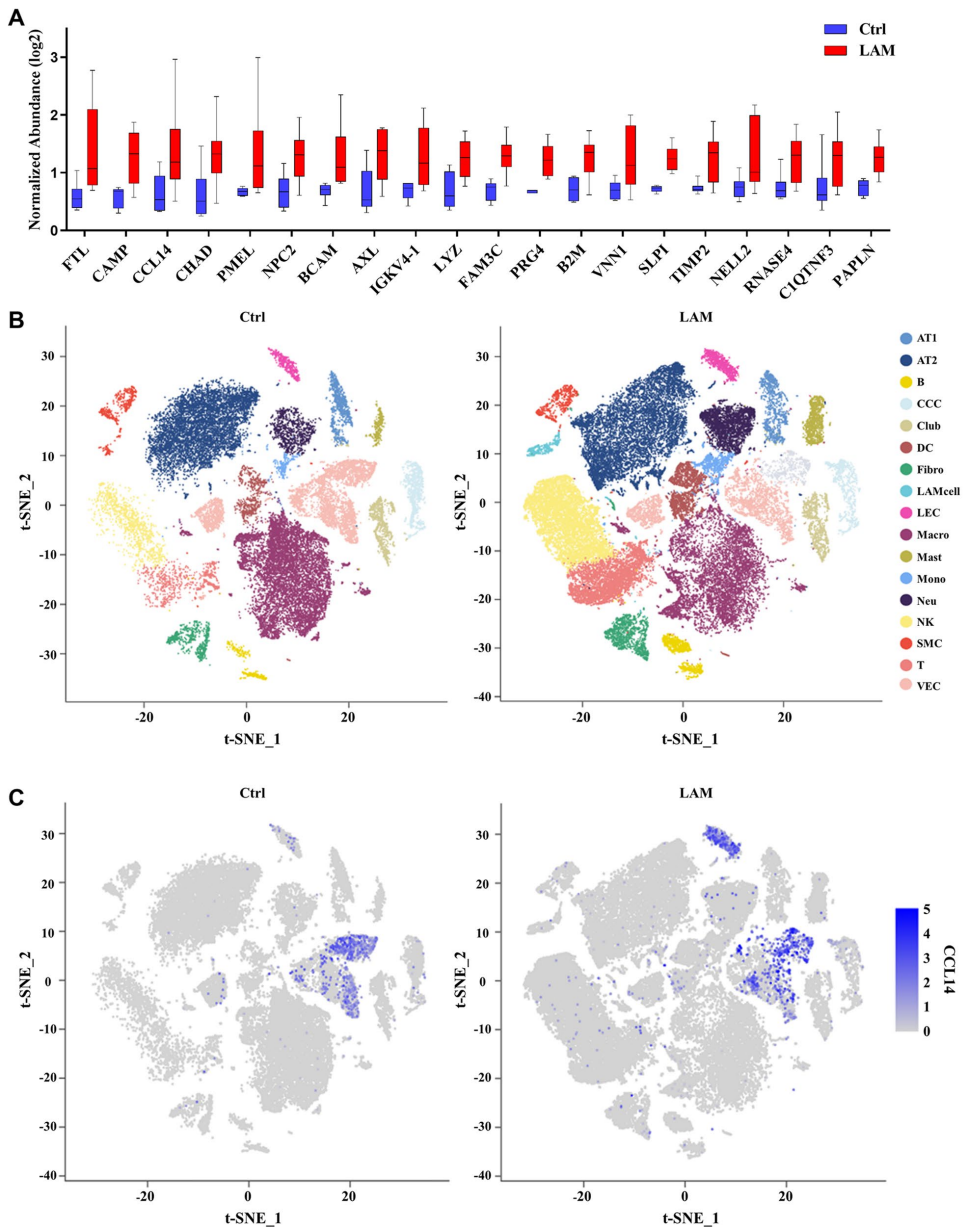

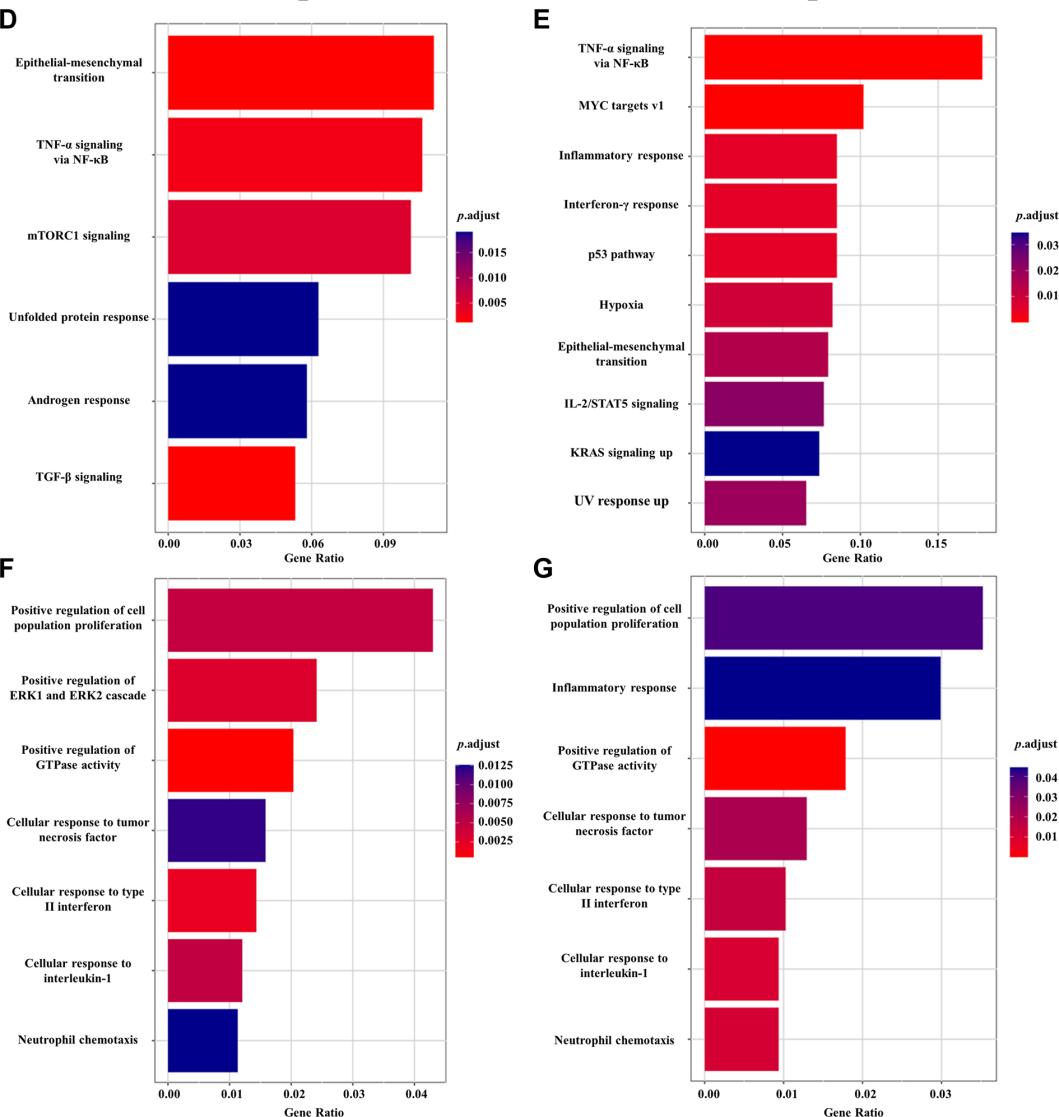
Proteomic and transcriptomic profiling reveals dysregulation of CCL14 in LAM. (**A**) top 20 significantly upregulated plasma proteins identified by proteomics comparing LAM patients to healthy controls. (**B**) t-SNE visualization of scRNA-seq data from 5 controls (right) and 6 LAM (left) lung tissues, annotated by cell-type-specific markers. AT1, alveolar type 1 cell. AT2, alveolar type 2 cell. B, B cell. CCC, ciliated columnar cell. Club, club cell. DC, dendritic cell. Fibro, fibroblast. LAM, LAM cell. LEC, lymphatic endothelial cell. Macro, macrophage. Mast, mast cell. Mono, monocyte. Neu, neutrophil. NK, natural killer cell. SMC, smooth muscle cell. T, T cell. VEC, vascular endothelial cell. (**C**) expression of CCL14 within the scRNA-seq t-SNE plots for donors (left) and LAM patients (right); color intensity reflects expression level. (**D-E**) GO-Biological processes enrichment of differentially expressed genes (DEGs) in LAM versus controls: (**D**) LECs; (**E**) VECs. (**F-G**) Hallmark pathway enrichment analysis of DEGs between CCL14+ and CCL14- subpopulations within LAM: (**F**) LECs; (**G**) VECs

Focusing on chemokines within our previously published plasma proteomics dataset of DEPs (16), we identified CCL14 as a prominent candidate. CCL14 demonstrated a 2-fold increase in LAM cohorts relative to healthy controls, ranking third among upregulated proteins; the top 20 upregulated proteins are presented in Fig. 1A.

To further investigate its cellular source and context, we integrated public pulmonary scRNA-seq datasets with our own scRNA-seq data comprising six LAM patients and five healthy donors. Unsupervised clustering partitioned the integrated dataset into 27 distinct cell clusters. Cellular distribution patterns were visualized via t-SNE. Annotation of these clusters was based on the expression of known cell type-specific marker genes (Additional file 1 Figure S1A, B; Fig. 1B). Consistent with the proteomic findings, transcriptomic analysis confirmed significantly higher CCL14 expression in the LAM group, predominantly localized to lymphatic endothelial cells (LECs) and vascular endothelial cells (VECs) (Fig. 1C).

GO enrichment analysis of differential genes comparing diseased and control LECs between disease and control LECs revealed that CCL14 is associated with positive regulation of cell population proliferation, cellular response to tumor necrosis factor, cellular response to type II interferon, neutrophil chemotaxis, etc. Similarly, comparative analysis of VECs identified CCL14 enrichment in positive regulation of cell population proliferation, inflammatory response, cellular response to interleukin-1, neutrophil chemotaxis, etc (Fig. 1D, E). To further investigate the functional impact of CCL14 expression within these cell types, we stratified LECs and VECs into CCL14-positive (CCL14+) and CCL14-negative (CCL14-) subpopulations. Subsequent Hallmark pathway enrichment analysis of differential genes between CCL14+ LECs and CCL14- LECs identified significant enrichment involved in epithelial mesenchymal transition, tumor necrosis factor-alpha (TNF-α) signaling via nuclear factor kappa-B (NFkB), mammalian target of rapamycin complex 1 (mTORC1) signaling, transforming growth factor-beta (TGF-β) signaling, etc. Likewise, comparison of CCL14+ VECs versus CCL14- VECs revealed enrichment in pathways including TNF-α signaling via NF-κB, MYC targets v1, interferon γ response, p53 pathway, etc (Fig. 1F, G).

### Validation in lung tissues and in vitro functional assay

**Fig. 2:**
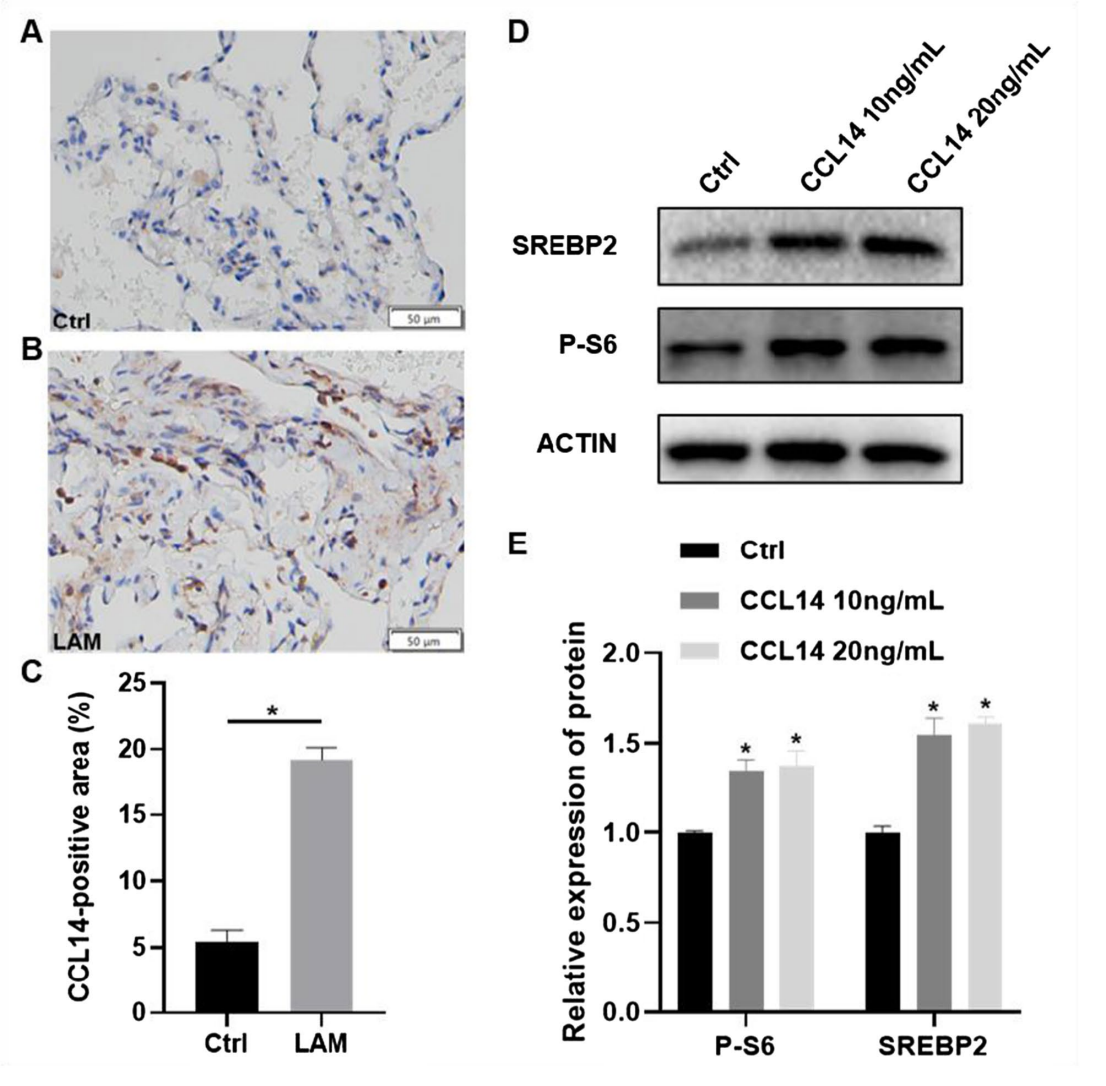
Validation of elevated CCL14 expression in LAM tissues and its functional activation of the mTOR pathway. (**A, B**) Representative images of immunohistochemical (IHC) staining for CCL14 in (**A**) control lung tissue and (**B**) LAM lung tissue. Scale bars, 50 µm. (**C**) quantitative analysis of the percentage of CCL14-positive area in lung tissues from 6 controls and 7 LAM patients (five random fields per sample were analyzed). Data are presented as mean ± SEM. (**D**) Representative Western blot images of key mTOR downstream proteins. (**E**) quantitative analysis of the protein expression levels. Data are presented as mean ± SD (*n* ≥ 3). *p < 0.05 vs. control group. P-S6, phosphorylation of S6 ribosomal protein. Ctrl, control

To empirically validate the proteomic and transcriptomic findings regarding CCL14, we first assessed its protein expression in human lung tissues. Immunohistochemical (IHC) analysis of tissues from 7 patients with LAM and 6 controls revealed a markedly elevated expression of CCL14 in LAM lesions compared to control tissues (Fig. 2A–C).

We next investigated the functional consequence of CCL14 upregulation by treating HUVECs with recombinant CCL14. Consistent with bioinformatic predictions implicating CCL14 in mTORC1 activation, western blot analysis confirmed that CCL14 significantly enhanced the phosphorylation of the S6 ribosomal protein (p-S6), a established downstream effector of mTORC1 signaling. We also observed upregulation of SREBP2, another transcriptional target of mTORC1 (Fig. 2D, E). In contrast, under the same experimental conditions, we detected no phosphorylation of P65, indicating that the NF-kB pathway was not activated (data not shown).

### CCL14-mediated intercellular signaling network in LAM

**Fig. 3:**
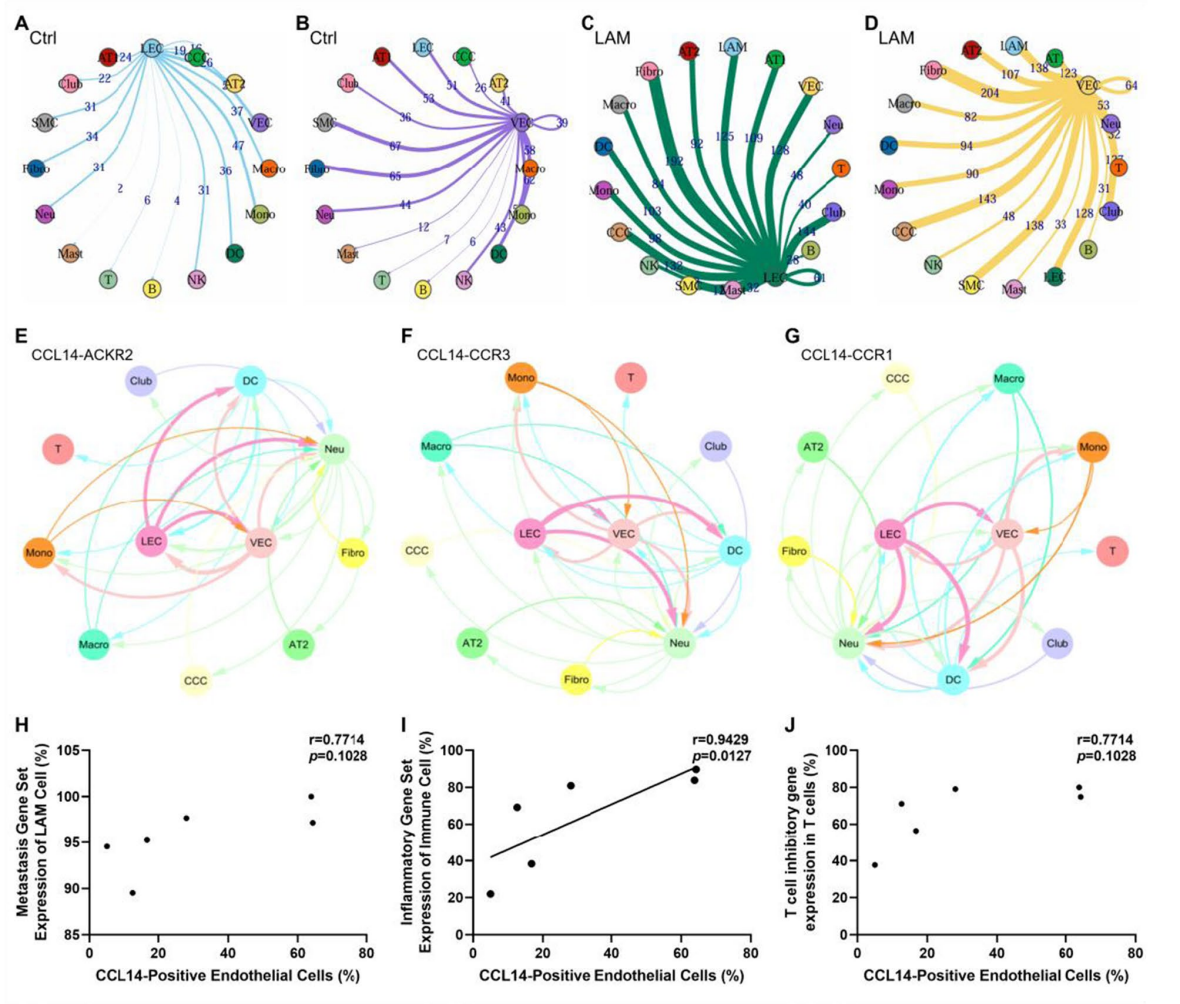
CCL14-mediated intercellular communication networks in LAM. (**A-D**) CellPhoneDB analysis of intercellular communication networks: (**A**) LECs and (**B**) VECs in controls; (**C**) LECs and (**D**) VECs in LAM. Line thickness indicates interaction robustness. (**E-G**) top 30 significant ligand-receptor interactions for (**E**) CCL14–ACKR2, (**F**) CCL14–CCR3 and (**G**) CCL14–CCR1 signatures in LAM. Edge width represents signaling strength. (**H-J**) correlation analyses in LAM samples between the proportion of CCL14+ endothelial cells and expression signatures of (**H**) LAM cell metastasis-associated gene signature, (**I**) immune cell inflammation gene signature and (**J**) T-cell inhibitory gene signature

CellPhoneDB analysis of intercellular communication networks revealed significant dysregulation in the LAM cohort compared to healthy controls. Specifically, LECs and VECs exhibited markedly enhanced interaction strength with other cell populations relative to healthy controls (Fig. 2A, B). Subsequent ligand-receptor screening within the disease cohort identified atypical chemokine receptor 2 (ACKR2), chemokine C-C-Motif receptor 3 (CCR3), and chemokine C-C-Motif receptor 1 (CCR1) as high-confidence receptors for the chemokine CCL14. Visualization of CCL14–ACKR2/CCR3/CCR1 networks demonstrated these interactions predominantly originate from LECs or VECs, targeting: innate immune cells (monocytes, dendritic cells, neutrophils), alveolar type II (AT2) cells, and bidirectional crosstalk between LECs and VECs (Fig. 3C–E). Additionally, scRNA-seq analysis of six LAM patients revealed that the proportion of CCL14+ endothelial cells (ECs) within the endothelial compartment exhibited a statistically significant positive correlation with the expression of inflammatory gene sets in immune cells (*r* = 0.9429, *p* = 0.0127) and a positive trend with the expression of metastasis-associated gene signatures in LAM cells (*r* = 0.7714, *p* = 0.1028) and T cell inhibitory signatures (*r* = 0.7714, *p* = 0.1028) (Fig. 3F–H). These findings implicate CCL14+ ECs as potential contributors to the formation of a pro-metastatic niche and immune evasion within the LAM tumor microenvironment.

### Association between CCL14 levels and clinical phenotypes

To further validate the aforementioned findings, we enrolled 53 LAM patients and 25 healthy controls. The clinical characteristics of the participants are summarized in Table 1. No significant differences in age or BMI were observed between the LAM group and the healthy controls. Within the LAM cohort, sporadic LAM accounted for 94%. The prevalence of pneumothorax, renal angiomyolipoma, retroperitoneal involvement, and chylothorax were 43, 28, 26, and 17%, respectively. At baseline, 40% were receiving rapamycin therapy, 49% exhibited Grade III cystic changes on chest CT, and the median FEV_1_% predicted and DLCO% predicted were 75.1% (IQR 55.7% to 91.6%) and 51.8% (IQR 35.0% to 72.5%), respectively.

**Table 1:** Baseline data of subjects

Characteristics	LAM (*n* = 53)	HC (*n* = 25)	*p* value
Age (year)	42 (35,48)	43 (32,51)	0.768
BMI (kg/m^2^)	21.5 (19.9,23.8)	22.2 (20.8,24.4)	0.203
Pneumothorax	23 (43)	0 (0)	-
Renal AML	15 (28)	0 (0)	-
Retroperitoneal lymphangioleiomyoma	14 (26)	0 (0)	-
Chylous effusion	9 (17)	0 (0)	-
TSC-LAM	3 (6)	0 (0)	-
Rapamycin treatment	21 (40)	0 (0)	-
CT grade III	26 (49)	0 (0)	-
FEV_1_%pred	75.1 (55.7,91.6)^&^	-	-
FVC%pred	98.1 (83.8,108.8)^&^	-	-
DLCO%pred	51.8 (35.0,72.5)^¶^	-	-
DLCO/VA%pred	62.6 (38.8,79.6)^¶^	-	-

Data are presented as median (IQR-25%, IQR-75%) or number (%). LAM, lymphangioleiomyomatosis; HC, healthy controls; BMI, body mass index; AML, angiomyolipoma; TSC, Tuberous Sclerosis Complex; FEV1, forced expiratory volume in 1s; FVC, forced vital capacity; DLCO, diffusing capacity of the lungs for carbon monoxide; DLCO/VA, ratio of carbon monoxide diffusion capacity to alveolar ventilation; pred, predicted. ^&^n=44, ^¶^n=41

**Fig. 4:**
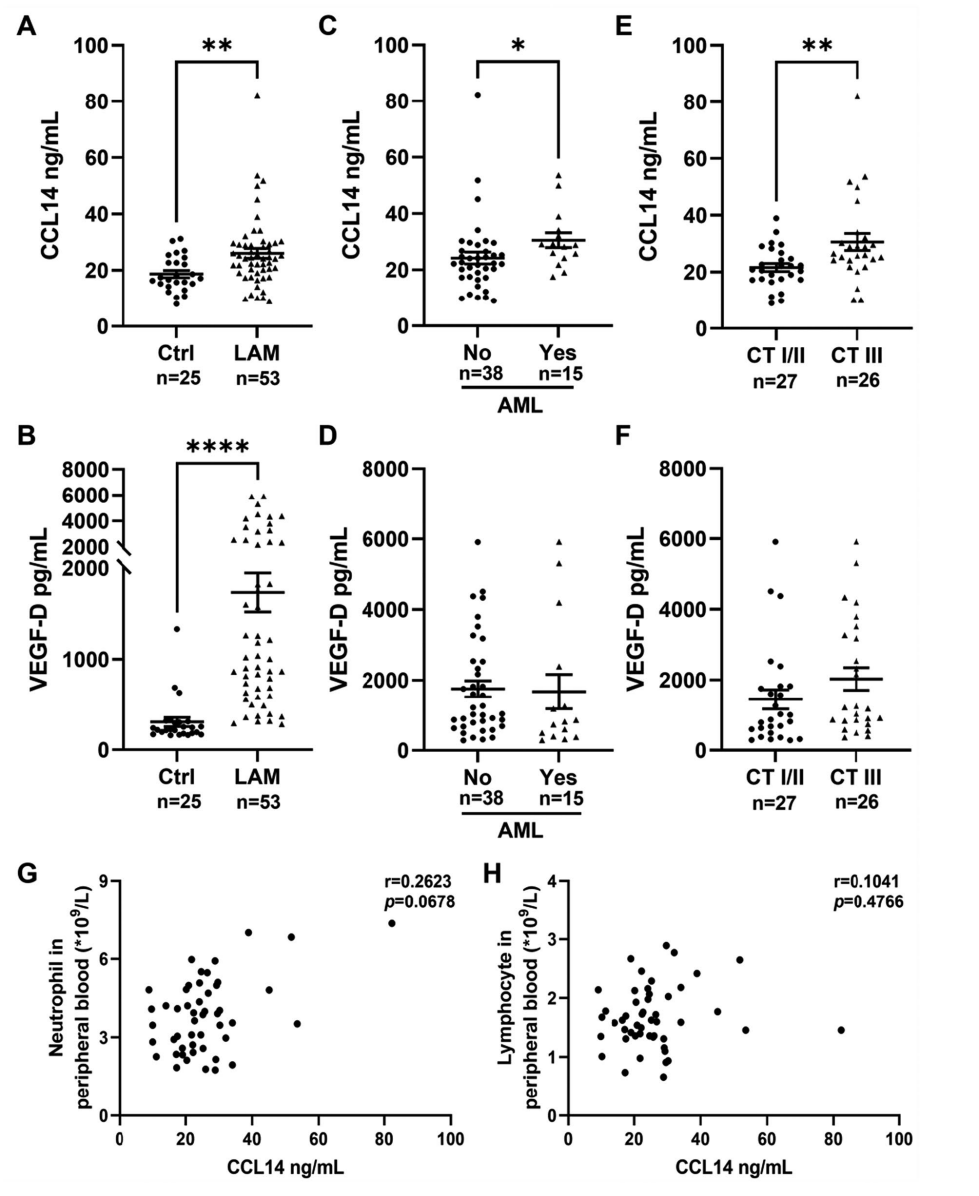
Correlation between CCL14 levels and clinical phenotypes. (**A**) plasma CCL14 concentrations and (**B**) serum VEGF-D levels in LAM patients (*n* = 53) versus healthy controls (*n* = 25). (**C**) CCL14 and (**D**) VEGF-D levels in LAM patients with versus without renal AMLs. (**E**) CCL14 and (**F**) VEGF-D concentrations between patients with CT grade I/II and grade III cystic lung changes. (**G-H**) correlation of CCL14 with (**G**) absolute peripheral neutrophil and (**H**) lymphocyte counts. *p < 0.05, **p < 0.01, ****p < 0.0001

ELISA analysis revealed significantly higher plasma CCL14 expression levels in the LAM group compared to the control group (*p* = 0.003). Notably, patients with renal angiomyolipoma demonstrated significantly higher CCL14 expression than those without renal involvement (*p* = 0.013). Similarly, patients classified as CT grade III cystic changes showed increased CCL14 expression relative to those with CT grade I or II (*p* = 0.006) (Fig. 4A–C). Consistent with previous studies, serum VEGF-D—a core diagnostic biomarker for LAM—were markedly elevated in the LAM patients. Nevertheless, in our cohort, no significant association was found between VEGF-D levels and either renal involvement status or CT grade severity (Fig. 4D–F). Additionally, plasma CCL14 levels showed no correlation with FEV_1_% predicted, DLCO% predicted, serum VEGF-D levels, history of pneumothorax, chylous effusion, retroperitoneal lymphangioleiomyoma, rapamycin therapy, or menopausal status (Additional file 1 Figure S2A-H). Further analysis indicated a positive correlation trend between plasma CCL14 levels and peripheral blood neutrophil count (*r* = 0.2623, *p* = 0.0678), whereas no significant correlation was observed with lymphocyte count (Fig. 4G, H).

### CCL14 as a predictive biomarker for LAM progression

**Fig. 5:**
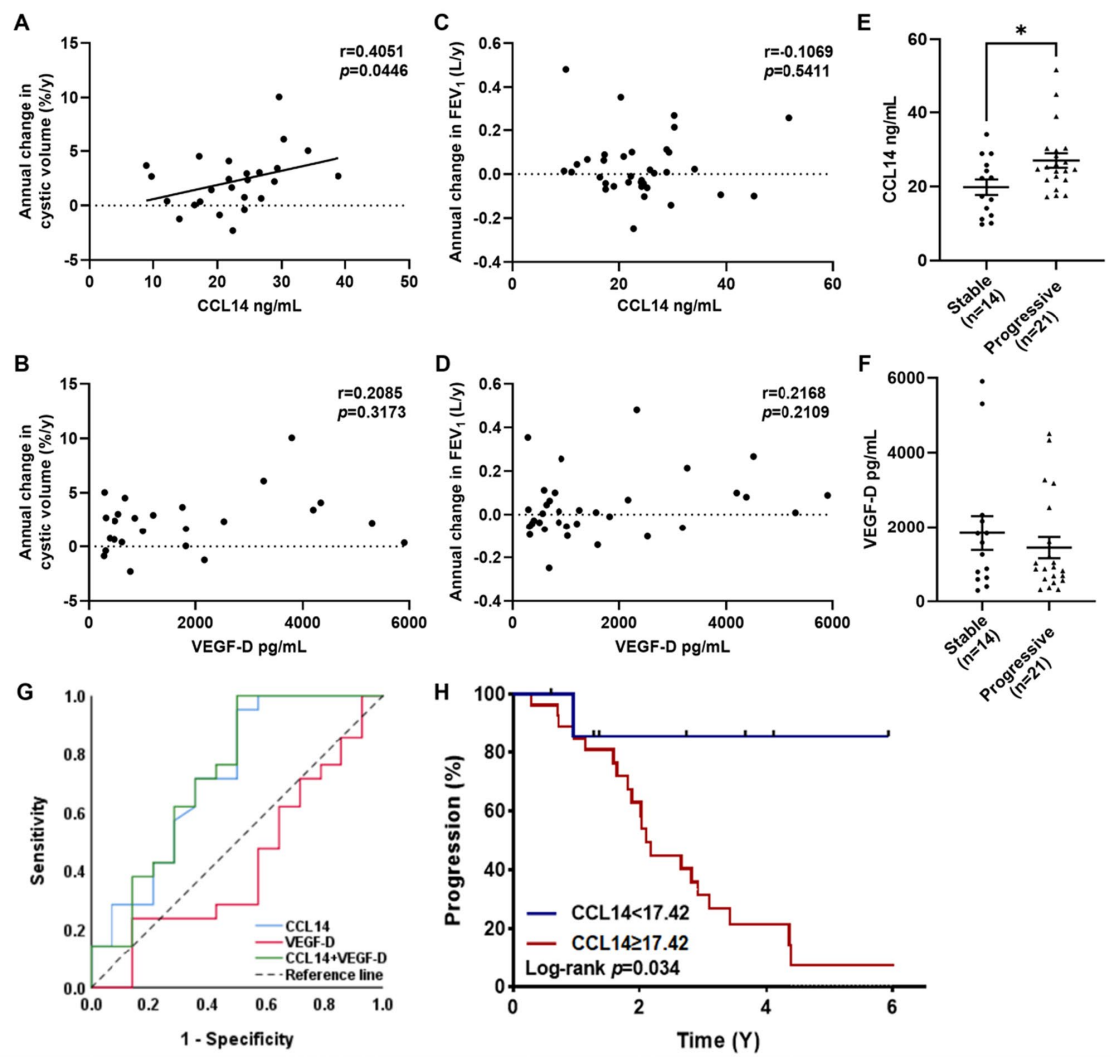
Association of circulating CCL14 and VEGF-D with disease progression. (**A-B**) association of baseline (**A**) CCL14 or (**B**) VEGF-D with annual cystic volume change (*n* = 25). (**C-D**) relationship of baseline (**C**) CCL14 or (**D**) VEGF-D to annual change in FEV_1_ (*n* = 35). (**E-F**) comparison of baseline (**E**) CCL14 or (**F**) VEGF-D concentrations between disease-stable and disease-progressive groups. (**G**) receiver operating characteristic (ROC) curves evaluating the predictive performance of CCL14, VEGF-D, and their combination for disease progression. (**H**) kaplan-meier analysis of disease progression comparing patients stratified by the CCL14 cutoff (≥17.42 vs. < 17.42)

Among the 53 patients enrolled at baseline, 25 cases had accessible ≥2 chest CT images (median follow-up: 2.2 years, IQR: 1.2–3.6 years), and 35 cases had ≥2 sets of pulmonary function test data (median follow-up: 1.6 years, IQR: 1.0–3.1 years). baseline plasma CCL14 levels exhibited a significant positive correlation with the annual percentage change in cystic lesion burden quantified by CT (*r* = 0.4051, *p* = 0.0446), whereas no statistically significant association was observed between CCL14 levels and the annual decline rate in FEV_1_ (*r* = 0.2058, *p* = 0.3173) (Fig. 5A, C). Disease progression status was evaluated in the 35 patients with longitudinal follow-up data using the predefined criteria detailed in the Methods section. As shown in Table 2, fourteen patients (40%) were categorized as stable (median follow-up: 2.1 years, IQR: 1.3–4.0 years) and twenty-one patients (60%) as progressive (median follow-up: 2.0 years, IQR: 1.1–2.8 years). The majority of patients in both cohorts received rapamycin therapy (stable group: 12/14; progressive group: 20/21), either initiated at baseline or during follow-up. Comparative analysis demonstrated that baseline CCL14 concentrations were significantly elevated in the progression group compared to the stable group (*p* = 0.0266) (Fig. 5E). We performed ROC curve analysis to evaluate the predictive power of CCL14 for disease progression. The area under the curve (AUC) for CCL14 alone was 0.723 (*p* = 0.027), indicating a statistically significant predictive value. A cutoff value of 17.42 was identified for CCL14, whereby patients with levels greater than or equal to this threshold were associated with a poorer disease prognosis (Fig. 5G, H).

**Table 2:** Clinical characteristics and longitudinal outcomes in stable vs progressive LAM patients

Characteristics	stable (n = 14)	progressive (n = 21)	P-value
**Age (year)**	45 (39, 49)	44 (37, 50)	0.914
**BMI (kg/m**^**2**^)	20.7 (19.2, 24.0)	22.7 (20.7, 24.0)	0.274
**Follow-up duration (year)**	2.1 (1.3, 4.0)	2.0 (1.1, 2.8)	0.606
**Pneumothorax**			
Baseline history	5 (36)	8 (38)	0.886
Refractory/recurrent (≥3 episodes/year)	0 (0)	4 (19)	-
**Renal AML**			
Baseline history	4 (29)	5 (24)	0.752
Annual growth ≥2 mm	0 (0)	0 (0)	-
**Retroperitoneal lymphangioleiomyoma**			
Baseline history	3 (21)	8 (38)	0.298
Annual growth ≥2 mm	0 (0)	0 (0)	-
**Chylous effusion**			
Baseline history	2 (14)	4 (19)	0.714
Refractory/progressive	0 (0)	1 (5)	-
**Rapamycin therapy**			
Patients treated	12 (86)	20 (95)	0.324
Treatment duration (year)	4.1 (1.5, 5.0)	3.4 (1.7, 5.1)	0.977
**Annual change in lung function**			
FEV_1_ (L/year)	0.02 (0.01, 0.08)	−0.05 (−0.09, −0.01) ^&^	0.004
DLCO (mmol/min/kpa/year)	0.08 (−0.01, 0.26)	−0.18 (−0.34, 0.01)^&^	0.006
Relative DLCO/VA (%/year)	0.8 (−1.41, 5.43)	−3.54 (−6.44, −1.17)^&^	0.008

Data are presented as median (IQR-25%, IQR-75%) or number (%). ^&^n=18. Relative DLCO/VA, annual percentage change in DLCO/VA relative to baseline

In parallel analyses, the established serum biomarker VEGF-D showed no significant correlation with either the annual change in cystic lesion burden (*r* = 0.2085, *p* = 0.3173) or FEV_1_ decline rate (*r* = 0.2168, *p* = 0.2109) (Fig. 5B, D). Furthermore, serum VEGF-D levels did not differ significantly between the stable and progressive disease groups (*p* = 0.4742) (Fig. 5F). The AUC for VEGF-D alone was 0.425 (*p* = 0.459), which was not statistically significant (Fig. 5G).

## Discussion

This integrative investigation employing plasma proteomics, single-cell transcriptomics, and clinical assessment identifies CCL14 as an important regulatory molecule. We provide the first demonstration that CCL14 is significantly elevated in LAM patients and mediates intercellular communication within this pathological niche. Moreover, CCL14 expression correlated significantly with disease severity severity, including renal angiomyolipoma and severe cystic lung destruction, and effectively predicted disease progression.

Prior studies have demonstrated that multiple chemokines, such as CCL2, CXCL12 and CXCL1, are dysregulated in LAM. These chemokines promote the migration and invasion of LAM cells, recruit immune cells such as mast cells, macrophages and neutrophils, and contribute to extracellular matrix remodeling and pulmonary cyst formation [6, 9, 22–26]. Extending these findings, our plasma proteomic profiling identified CCL14 as a significantly upregulated chemokine in LAM patients compared to controls. Single-cell transcriptomic profiling localized predominant CCL14 expression to LECs and a VECs, cell types critically implicated in LAM progression through lymphangiogenesis and angiogenesis. Functional enrichment further linked CCL14 to proliferative pathways (mTORC1, MYC targets), pro-inflammatory signaling (TNF-α/NF-κB), and chemotaxis (recruitment of neutrophils, monocytes, and lymphocytes). These functions align with established hallmarks of LAM: including excessive LAM cell proliferation driven by mTOR dysregulation, chronic inflammation-mediated tissue remodeling, and immune cell infiltration leading to parenchymal destruction [9, 24–27]. Consistent with its bioinformatic association with mTORC1 signaling, our in vitro experiments demonstrated that recombinant CCL14 activated the mTOR pathway in HUVECs. CellPhoneDB-based ligand-receptor interaction analyses further revealed the cellular communication network mediated by CCL14 in LAM, with LECs and VECs identified as the principal cellular sources of CCL14 signaling and ACKR2, CCR3, and CCR1 as high-confidence receptors expressed on immune cells (monocytes, neutrophils and dendritic cells), AT2 cells, and LECs/VECs themselves. This suggests that CCL14 may shape the LAM tumor microenvironment through recruiting immune cells, interacting with alveolar epithelium, facilitating autocrine/paracrine signaling among endothelial cells. Supporting this, further correlation analyses indicate that the percentage of CCL14+ endothelial cells associates with gene sets related to LAM cell metastasis, immune-inflammatory responses, and T-cell immunosuppression,, implying a potential role for CCL14 in promoting metastatic potential, inflammatory responses, and fostering an immunosuppressive niche within the LAM lesion. CCL14 signaling exerts divergent, context-dependent effects: in liver cancer, a solid tumor, it inhibits tumor growth and promotes apoptosis through inhibiting Wnt/β-catenin pathway [13, 17], whereas in the inflammatory LAM microenvironment, it may promote lymphangiogenesis and disease progression. While CCL14 can bind multiple receptors (e.g., CCR1, CCR3, CCR5), its dominant receptor in specific cancers remains elusive [13, 18].

In the clinical cohort, we confirmed significantly elevated plasma CCL14 levels in LAM patients compared to controls, with higher concentrations observed in patients presenting with renal AMLs or more severe CT grade. This implicates that CCL14 may serve as a potential biomarker reflecting disease burden, parenchymal destruction and structural damage in LAM. However, the absence of a significant correlation between CCL14 levels and FEV_1_% predicted or DLCO% predicted requires careful consideration. Although lung function parameters serve as established metrics for quantifying functional impairment in LAM, their dissociation from CCL14 levels likely reflects distinct underlying pathological processes. CCL14 may predominantly indicate structural damage (cyst formation and tissue remodeling) rather than immediate functional compromise. These architectural changes accumulate progressively and may reach a substantial threshold before manifesting as functional impairment measurable by pulmonary function tests. Additionally, this analysis could be constrained by the missing pulmonary function data from severe patients who could not tolerate the tests. This selection bias might have truncated the functional dataset, effectively excluding individuals with poor lung function. Consequently, our analysis may underestimate the true correlation between CCL14 and functional impairment. Critically, our findings highlight CCL14 as a potential prognostic indicator: baseline CCL14 levels were higher in patients with disease progression compared to those with stable disease. Furthermore, CCL14 showed a positive correlation with annual increases in the percentage of pulmonary cystic volume on CT, validating its utility as a predictor for progressive structural damage.

Contrasting with CCL14, the established biomarker VEGF-D showed no association with AMLs or CT severity in our cohort. Previous studies have reported divergent findings regarding VEGF-D’s relationship with AMLs, including positive correlations, negative correlations, or no association between VEGF-D levels and AMLs presence or size in LAM patients [4, 5, 28–30]. Xu et al. proposed that AMLs size, rather than mere presence, might correlate more logically with VEGF-D levels [5]. Discrepancies with earlier reports of VEGF-D correlating with CT grade may arise from patient heterogeneity—differences in the proportion of patients receiving rapamycin treatment at baseline or presenting with lymphatic involvement, factors potentially affecting VEGF-D expression [5, 31–33]. Regarding functional parameters, several studies and our prior findings have reported an inverse relationship between VEGF-D levels and DLCO% predicted, DLCO/VA% predicted, FEV_1_ or FEV_1_/FVC, supporting the role of VEGF-D as a biomarker indicating functional impairment [4, 5, 16, 32]. In terms of prognosis, longitudinal assessment revealed no significant difference in baseline VEGF-D levels between patients with progressive and stable groups. Similarly, no correlations were observed between baseline VEGF-D levels and annual changes in either pulmonary cystic volume percentage or FEV_1_, despite prior reports indicating an inverse relationship between VEGF-D and the rate of FEV_1_ decline [4, 21]. Collectively, these findings suggest that CCL14 and VEGF-D reflect distinct aspects of LAM pathogenesis: CCL14 appears more closely linked to structural progression (disease burden, cystic destruction), while VEGF-D primarily reflects functional impairment and lymphatic pathophysiology.

There are several limitations to the study. Firstly, the predicted ligand-receptor interactions between CCL14 and its candidate receptors (ACKR2, CCR1, CCR3) are primarily derived from bioinformatic analyses. These require functional confirmation through in vitro co-culture assays or in vivo models. Secondly, the single-center cohort and restricted sample size of our clinical cohort may diminish statistical power and introduce selection bias, thereby limiting generalizability. Future large-scale, multi-center prospective studies are required to verify these findings. Thirdly, while our findings suggest CCL14 holds promise as a predictor of disease progression, the assessment of progression was conducted within a relatively limited follow-up period. Thus, the long-term prognostic value of CCL14 requires further validation.

## Conclusions

This study demonstrates that CCL14 is a critical mediator of intercellular crosstalk within the LAM tumor microenvironment. Predominantly derived from LECs and VECs, CCL14 could modulate critical biological processes such as proliferation, inflammation, and metastatic niche formation. Clinically, CCL14 levels correlate with disease severity and predict future progression independently of VEGF-D, highlighting its value as a complementary biomarker for LAM management. Collectively, these findings provide new mechanistic insights into LAM pathogenesis, identifying CCL14 as both a potential biomarker and therapeutic target.

## Electronic supplementary material

Below is the link to the electronic supplementary material.

Supplementary Material 1

Supplementary Material 2

Supplementary Material 3

## Data Availability

The datasets used and/or analysed during the current study are available from the corresponding author on reasonable request.

## Abbreviations

ACKR2: Atypical chemokine receptor 2
AMLs: Angiomyolipomas
AT2: Alveolar type II
ATS/JRS: American Thoracic Society/Japanese Respiratory Society
AUC: Area under the curve
CCL14: C-C motif chemokine ligand 14
CCR3: Chemokine C-C-Motif receptor 3
CCR1: Chemokine C-C-Motif receptor 1
CT: Computed tomography
DEPs: Differentially expressed proteins
DLCO: Diffusing capacity of the lungs for carbon monoxide
DLCO/VA: Ratio of carbon monoxide diffusion capacity to alveolar ventilation
ECs: Endothelial cells
FEV_1_: Forced expiratory volume in 1s
FVC: Forced vital capacity
GO: Gene Ontology
HUVECs: Human Umbilical Vein Endothelial Cells
IHC: Immunohistochemical
LAM: Lymphangioleiomyomatosis
LECs: Lymphatic endothelial cells
mTOR: Mammalian target of rapamycin
mTORC1: Mammalian target of rapamycin complex 1
NFkB: Nuclear factor kappa-B
P-S6: Phosphorylation of S6 ribosomal protein
ROC: Receiver operating characteristic
scRNA-seq: Single-cell RNA sequencing
TME: Tumor microenvironment
TGF-β: Transforming growth factor-beta
TNF-α: Tumor necrosis factor-alpha
t-SNE: T-distributed stochastic neighbor embedding
VEGF-D: Vascular endothelial growth factor D
VECs: Vascular endothelial cells
