# Supplementary figures and images for: CCL14, identified by multi-omics approach, serves as a novel indicator of disease severity and progression in lymphangioleiomyomatosis
Source: Orphanet J Rare Dis. 2026 Jan 20;21:59. doi: 10.1186/s13023-025-04193-2 (PMC12905834; doi:10.1186/s13023-025-04193-2)

Additional file 1

Figure S1

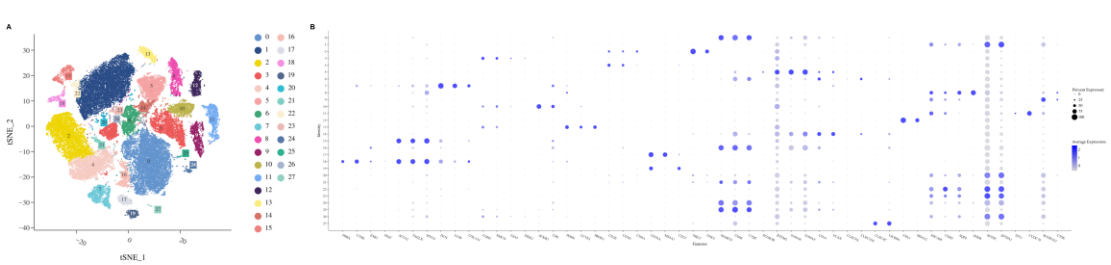

Figure S2

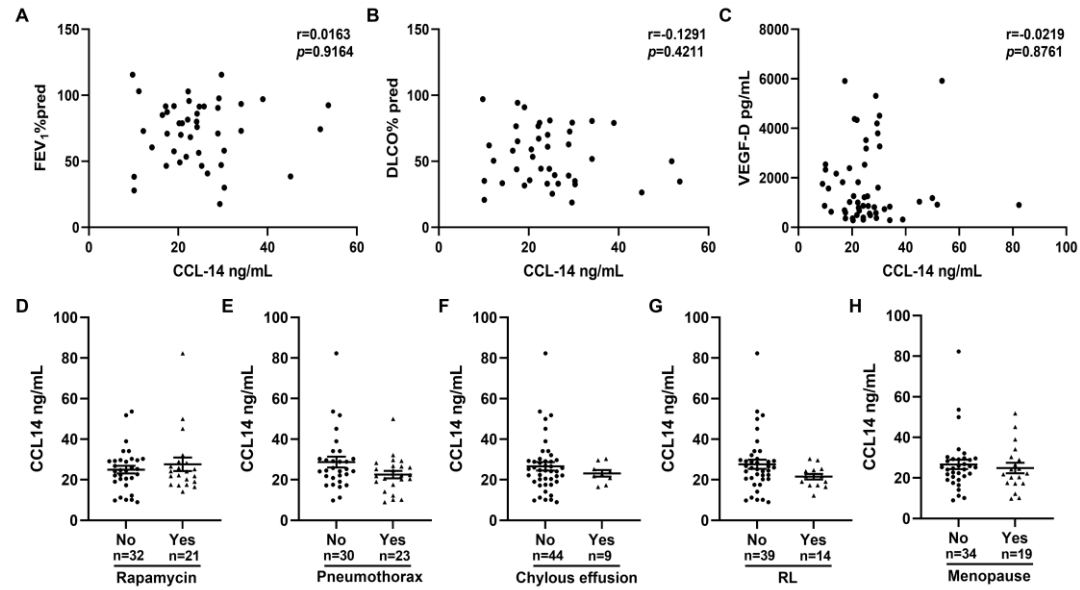

Supplement: Supplementary file 1 — Supplementary Material 1 [file 13023_2025_4193_MOESM1_ESM.pdf]

Additional file 2

Figure S1

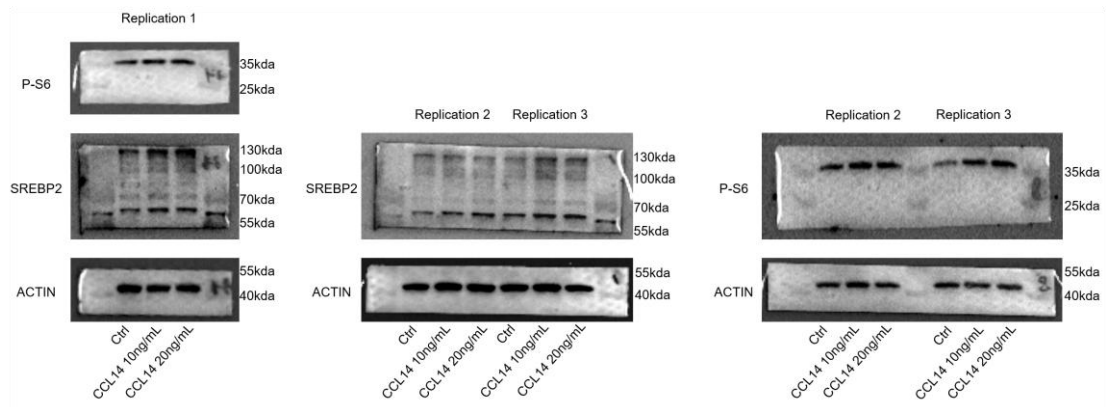

Supplement: Supplementary file 2 — Supplementary Material 2 [file 13023_2025_4193_MOESM2_ESM.pdf]
