# Supplementary material for: CCL14, identified by multi-omics approach, serves as a novel indicator of disease severity and progression in lymphangioleiomyomatosis
Source: Orphanet J Rare Dis. 2026 Jan 20;21:59. doi: 10.1186/s13023-025-04193-2 (PMC12905834; doi:10.1186/s13023-025-04193-2)
Supplement: Supplementary file 3 — Supplementary Material 3 [file 13023_2025_4193_MOESM3_ESM.docx]

Additional file 1

Figure S1. scRNA-seq clustering and annotation. (A) Cellular clusters identified by scRNA-seq in 6 LAM patients and 5 donors. (B) Dot plot visualization of cluster-specific marker expression: dot size corresponds to fraction of cells expressing marker genes; color intensity represents average expression level.

Figure S2. CCL14 expression across LAM clinical phenotypes. (A-C) Association between CCL14 and (A) FEV_1_%pred (n=44), (B) DLCO%pred (n=41), and (C) VEGF-D (n=53). pred, predicted. (D-H) Comparable CCL14 levels stratified by: (D) rapamycin exposure, history of (E) pneumothorax, (F) chylous effusion or (G) RL, (H) menopausal status. RL, retroperitoneal lymphangioleiomyoma.

Additional file 2

Figure S1. Original blot images for SREBP2, P-S6 and ACTIN (molecular weight markers at right). Experiments were performed in triplicate.
